# Screening and Identification of a Novel Anti-tuberculosis Compound That Targets Deoxyuridine 5′-Triphosphate Nucleotidohydrolase

**DOI:** 10.3389/fmicb.2021.757914

**Published:** 2021-10-11

**Authors:** Yu Zhang, Hongjuan Zhang, Ying Chen, Luyao Qiao, Yanxing Han, Yuan Lin, Shuyi Si, Jian-Dong Jiang

**Affiliations:** ^1^State Key Laboratory of Bioactive Substances and Function of Natural Medicine, Institute of Materia Medica, Chinese Academy of Medical Sciences and Peking Union Medical College, Beijing, China; ^2^Institute of Medicinal Biotechnology, Chinese Academy of Medical Sciences and Peking Union Medical College, Beijing, China

**Keywords:** anti-tuberculosis, dUTPase, high-throughput screening, inhibitor, molecular docking

## Abstract

Tuberculosis (TB) is still a threat to humans worldwide. The rise of drug-resistant TB strains has escalated the need for developing effective anti-TB agents. Deoxyuridine 5′-triphosphate nucleotidohydrolase (dUTPase) is essential for thymidylate biosynthesis to maintain the DNA integrity. In *Mycobacterium tuberculosis*, dUTPase provides the sole source for thymidylate biosynthesis, which also has the specific five-residue loop and the binding pockets absent in human dUTPase. Therefore, dUTPase has been regarded as a promising anti-TB drug target. Herein, we used a luminescence-based dUTPase assay to search for the inhibitors target *M. tuberculosis* dUTPase (Mt-dUTPase) and identified compound F0414 as a potent Mt-dUTPase inhibitor with an IC_50_ of 0.80 ± 0.09 μM. F0414 exhibited anti-TB activity with low cytotoxicity. Molecular docking model and site-directed mutation experiments revealed that P79 was the key residue in the interaction of Mt-dUTPase and F0414. Moreover, F0414 was shown to have stronger binding with Mt-dUTPase than with Mt-P79A-dUTPase by surface plasmon resonance (SPR) detection. Interestingly, F0414 exhibited insensitivity and weak directly binding on human dUTPase compared with that on Mt-dUTPase. All the results highlight that F0414 is the first compound reported to have anti-TB activity by inhibiting Mt-dUTPase, which indicates the potential application in anti-TB therapy.

## Introduction

Tuberculosis (TB) is a chronic infectious disease caused by *Mycobacterium tuberculosis (M. tuberculosis)*, which is the top cause of death from infectious disease [World Health organization, 2020a]. It has been a huge threat to public health worldwide for a long time, with about 10 million people infected each year [World Health organization, 2020b]. The emergence of multidrug-resistant TB (MDR-TB), extensively drug-resistant TB (XDR-TB), and HIV co-infection made it more challenging to prevent the prevalence of TB infections (Seung et al., 2015; Bell and Noursadeghi, 2018).

Traditional anti-TB drugs such as isoniazid, rifampicin, ethambutol, and pyrazinamide have been considered to be the front line in the battle against TB for many years (Suárez et al., 2019). After a long time of inactivity, increasing numbers of new anti-TB drugs have been seen in recent years (Singh et al., 2020). Both bedaquiline and delamanid are new anti-TB drugs approved in almost half a century (Pym et al., 2016; Liu et al., 2018; Li et al., 2019). However, with the increasing use in clinic, the resistance toward bedaquiline and delamanid appeared (Liu et al., 2018; Degiacomi et al., 2020). Some drug candidates are also in phase II and phase III clinical trials, such as SQ109, pretomanid (PA-824), telacebec (Q203), and so on (Pstragowski et al., 2017; de Jager et al., 2020; Deb and Biswas, 2021). Although dramatic progresses have been achieved, there is still an urgent need for new anti-TB agents that are highly active with low toxicity due to the increasing drug resistance problems. Therefore, novel drug targets for developing new anti-TB drugs are still necessary.

Deoxyuridine 5′-triphosphate nucleotidohydrolase (dUTPase) is a nucleotide metabolism–involved enzyme, which could catalyze the hydrolysis of dUTP to dUMP and inorganic pyrophosphate (PPi) (Ladner, 2001). Subsequently, dUMP was used as the substrate for dTMP. In the process of DNA synthesis, the dTMP will be transformed into dTTP ultimately (Vértessy and Tóth, 2009; Villela et al., 2011). As DNA polymerase does not distinguish between dTTP and dUTP, the synthesized DNA might have misincorporation by introducing dUTP instead of dTTP (Olinski et al., 2010). Therefore, dUTPase has an essential role in DNA synthesis by decreasing the intracellular dUTP/dTTP ratio and obstructing dUTP misincorporation into DNA.

dUTPase exists in both prokaryotes and eukaryotes, playing a critical role in cell metabolism (Yokogawa et al., 2020). Interestingly, in addition to the monofunctional dUTPase, it has only been reported that a bifunctional dCTP deaminase/dUTPase was also encoded in *M. tuberculosis* that could catalyze both the dCTP deamination and the dUTP hydrolysis (Pecsi et al., 2012). In contrast, humans encode thymidine kinase and the dCMP deaminase that have the two alternative dUTPase-mediated pathways (Vértessy and Tóth, 2009). Scientists also found that *M. tuberculosis* encoded dUTPase with a specific loop absent in human dUTPase (Varga et al., 2008; Pecsi et al., 2012). Based on the structural and functional differences of dUTPase between *M. tuberculosis* and human, it might be a potential drug target for anti-TB drug design.

There have been some dUTPase inhibitors reported, most of which are analogs of deoxyuridine triphosphate, such as TAS-114, 5′-tritylated deoxyuridine analogs, and β-branched acyclic nucleoside analogs (Nguyen et al., 2006; Baragaña et al., 2011; Yano et al., 2018). Only few researches focused on the dUTPase inhibitors with different structures from deoxyuridine triphosphate analogs. Lima et al. (2018) found that LabMol-144 and LabMol-146 had potential inhibition against *Plasmodium falciparum* dUTPase by virtual screening from the compound library. However, there are no dUTPase inhibitors of pathogenic bacteria reported.

In this research, we focused on screening inhibitors of *M. tuberculosis* dUTPase (Mt-dUTPase). First, we established the high-throughput screening model for inhibitors of Mt-dUTPase. Through screening for 5,000 compounds, we found that compound F0414 could inhibit Mt-dUTPase with an IC_50_ of 0.80 ± 0.09 μM. Then, we analyzed the binding mode of F0414 and Mt-dUTPase by molecular docking. P79 was the key amino acid in the binding between F0414 and Mt-dUTPase, further identified by activity analysis of mutant proteins and surface plasmon resonance (SPR) experiment. Interestingly, F0414 had a weaker inhibitory effect (IC_50_ = 3.18 ± 0.96 μM) on human dUTPase and showed weak binding with it observed by SPR. More importantly, F0414 had anti-TB activity *in vitro*, with little toxicity on mammalian cells. All these indicated that F0414 could selectively inhibit Mt-dUTPase and show potent anti-TB activity.

## Materials and Methods

### Materials

The restriction endonuclease *Nde* I and *Bam*H I were purchased from New England Biolabs (MA, United States). IPTG (isopropyl-β-D-thiogalactoside) was purchased from Sigma (MI, United States). dUTP was purchased from Takara (Kyoto, Japan). PPiLight inorganic pyrophosphate assay kit was purchased from Lonza (Basel, Switzerland). DMEM, DPBS, and other reagents used in the cell experiments were bought from Thermo Fisher (MA, United States). CM5 sensor chip and reagents used in SPR assays were bought from GE Healthcare (Uppsala, Sweden). The compound library was a commercial synthetic and natural product provided by Enamine (Kyiv, Ukraine) and was bought from the J&K Chemical company (Beijing, China). Molecular docking software Discovery Studio 2018 R2 was provided by Accelrys (CA, United States).

### Expression and Purification of *Mycobacterium tuberculosis* Deoxyuridine 5′-Triphosphate Nucleotidohydrolase

Mt-dUTPase gene was amplified through PCR, using H37Rv genome as the template. Primers were designed by Primer 5. Sense: 5′-TCATATGTCGACCACTCTGGCGATCGT-3′ (*Nde* I); antisense: 5′-CGGGATCCTTACAAACTCGCATGTCCG-3′ (*Bam*HI). The target gene was cloned into pET16b vector with 10× His tag on the N-terminal. Then the recombinant plasmid pET16b-Mt-dUTPase was transformed into *E*scherichia *coli* BL21 (DE3) for Mt-dUTPase protein expression.

The positive clone was grown at 37°C for 12 h in LB medium with ampicillin of 100 μg/ml. Then 0.5 mM IPTG was added to the culture at 30°C for 8 h. After that, cells were collected and the supernatant was loaded on the Ni^2+^ HisTrap chelating column. The loading buffer was 20 mM Na_3_PO_4_, 0.5 M NaCl, 30 mM imidazole, and pH 7.5 and the elution buffer was 20 mM Na_3_PO_4_, 0.5 M NaCl, 350 mM imidazole, and pH 7.5. The Mt-dUTPase protein was eluted by a stepwise gradient of imidazole in the elution buffer. The eluted samples were analyzed by SDS-PAGE and western blot (anti-His tag). The purified Mt-dUTPase concentration was measured by Bradford method. Samples were stored at −80°C.

### *Mycobacterium tuberculosis* Deoxyuridine 5′-Triphosphate Nucleotidohydrolase Activity Assay

dUTPase catalyzes the hydrolysis of dUTP to dUMP and PPi. The *in vitro* catalytic activity of dUTPase was detected by a PPi Light inorganic pyrophosphate assay kit. All the reaction components were added to a 96-well white plate, including 2 μl 40 ng/ml Mt-dUTPase, 36 μl reaction buffer (25 mM MOPS, pH 8.0, 10 mM KCl, 1.25 mM MgCl_2_, 0.1 mg/ml BSA, 0.005% Triton X, and 20% glycerol), and 2 μl 10 μM dUTP. Reagents in the PPi Light kit were diluted by the reaction buffer in a ratio of 1:4 prior to use. After 120 min of incubation at room temperature, the luminescence intensity of the reaction was measured by a microplate luminometer at 560 nm.

### Identification of *Mycobacterium tuberculosis* Deoxyuridine 5′-Triphosphate Nucleotidohydrolase Inhibitors

According to the Mt-dUTPase activity assay method, we established the high-throughput screening assay to screen a library of 5,000 compounds for the Mt-dUTPase inhibitors. The compound library was a commercial product, including some synthetic and natural product agents. Chemical compounds made through chemical process or produced by a living organism were included. In the initial screening, all the compounds were screened at a concentration of 10 μM. Mt-dUTPase was first incubated with the compound at room temperature for 1 h. Then the activity of Mt-dUTPase was determined as described previously. The inhibition rate of compound to the enzyme was calculated using the equation [(ΔS_*N*_ − ΔS_*S*_)/(ΔS_*N*_ − ΔS_*B*_)] × 100%, where ΔS_*N*_ is the intensity value of negative control treated with DMSO, ΔS_*S*_ refers to that of the sample group, and ΔS_*B*_ represents that of the blank control without the addition of Mt-dUTPase. The compound with more than 50% inhibition was regarded as the initial positive one. In the second screening, the initial positive compounds (10 μM) were incubated without Mt-dUTPase followed by the same activity assay method. At the same time, the cytotoxicity of the initial positive compounds at 10 μM were detected using the Cell Counting Kit-8 (CCK-8) assay. Then different concentrations of the initial positive compounds were applied. The inhibition curve was fitted by GraphPad Prism 8.0 software and IC_50_ was calculated. Trt-dU, a structure analog of dUTP, was used as the positive reference compound to test the Z′ factor for the reliability of the high-throughput screening assay (Nguyen et al., 2005; Whittingham et al., 2005). Z′ factor was calculated using the equation Z′ = 1 − 3(S_P_ + S_N_)/| μ_P_ − μ_N_|, where S_P_ and μ_P_ are the SDs and mean values of wells treated with 20 μM Trt-dU, respectively, and S_N_ and μ_N_ refer to that of wells treated with 2% DMSO.

### *In vitro* Anti-tuberculosis Activity

The microplate alamar blue assay (MABA) method was used to determine the anti-TB activity of compound F0414 (Cho et al., 2015). *M. tuberculosis* standard strain H37Rv, sensitive strain STB-FJ05349 and STB-FJ05060, multidrug-resistant strain MDR-FJ05120 and MDR-FJ05189, and extensive drug-resistant strain XDR-FJ05195 and XDR-XZ06008 were selected. The strains were inoculated into 96-well plates at a concentration of 10^6^ CFU/ml, then different concentrations (0.25–64 μg/ml) of F0414 were added. The first-line anti-TB drugs (isoniazid and rifampicin) were used as the positive controls. After 7 days of incubation at 37°C, the mixture of 20 μl 10× alamar blue and 50 μl 5% Tween-80 were added to observe the color change. MIC is defined as the minimum compound concentration of the well varying from blue to pink.

### Molecular Docking Between F0414 and *Mycobacterium tuberculosis* Deoxyuridine 5′-Triphosphate Nucleotidohydrolase

Discovery Studio 2018R2 was used to analyze the binding mode between F0414 and Mt-dUTPase using C-DOCKER program. The active center of Mt-dUTPase (PDB ID:1SIX) is defined as the receptor cavity (D24, D28, R149, Q113, and D83) with a radius of 10. Different conformations of F0414 were generated after optimization. According to the energy score and binding type, the docking mode and the key amino acids were determined.

### *Mycobacterium tuberculosis* Deoxyuridine 5′-Triphosphate Nucleotidohydrolase Mutations and Human Deoxyuridine 5′-Triphosphate Nucleotidohydrolase

Based on the molecular docking results, we mutated the key amino acid of Mt-dUTPase to further analyze the inhibitory mechanism of F0414. Meanwhile, an amino acid located outside of the active center was mutated for control. The expression, purification, and activity measurement of Mt-dUTPase mutant proteins were using the same method as that of Mt-dUTPase.

To identify the specificity of compound F0414 toward Mt-dUTPase, we also expressed and purified the human dUTPase. Template used in the PCR was cDNA of HEK293 cells. The primers were 5′-TCATATGTCGACCACTCTGGCGATCGT-3′ (sense, *Nde*I) and 5′-CGGGATCCTTACAAACTCGCATGTCCG-3′ (antisense, *Bam*HI). The target gene was also inserted into vector pET16b with 10× His tag on the N terminus. The protocols for expression, purification, and activity measurement of human dUTPase were the same as described previously.

### Interaction Between F0414 and *Mycobacterium tuberculosis* Deoxyuridine 5′-Triphosphate Nucleotidohydrolase Detected by Surface Plasmon Resonance

The SPR technology could be used to detect interactions between compounds and proteins. It was performed using a BIAcore T200 System. Mt-dUTPase, mutant Mt-dUTPase, and human dUTPase proteins were diluted by 10 mM sodium acetate (pH 4.5) and then was coated onto a CM5 sensor chip, respectively. Compound F0414 (0–200 μM) passed over the chip surface and the response units (RUs) were recorded in time. The equilibrium dissociation constant (*K*_*D*_) was determined using the BIAcore evaluation software.

### Cytotoxicity of Compound F0414

The cytotoxicity of F0414 was determined by CCK-8. MRC-5, Vero E6, and Caco-2 cells were tested. Concentrations of F0414 varied from 6.25 to 200 μM. Meanwhile, 2% DMSO was used as a negative control. After incubation for 24 h, 2-(2-methoxy-4-nitrophenyl)-3-(4-nitrophenyl)-5-(2,4-disulfophenyl)-2H-tetrazolium, monosodium salt (WST-8) was added. The experimental method referred to the kit instructions. All the experiments were repeated three times.

## Results

### Purification and Characterization of *Mycobacterium tuberculosis* Deoxyuridine 5′-Triphosphate Nucleotidohydrolase

**FIGURE 1:**
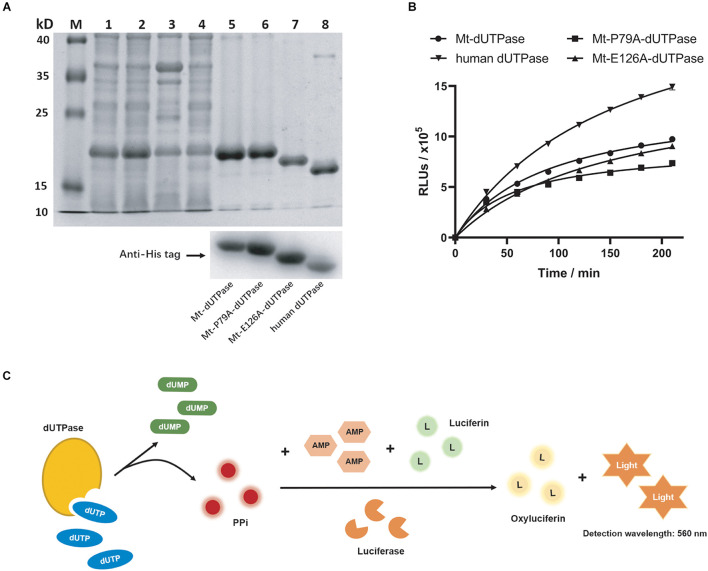
Purification and characterization of dUTPase. **(A)** Identification of dUTPase by SDS-PAGE (top) and western blot (bottom). Lane M, protein ladder; lane 1, total proteins of *E*. *coli* BL21 (DE3) containing Mt-dUTPase; lane 2, the supernatants of lytic *E. coli* BL21 (DE3) containing Mt-dUTPase; lane 3, the sediment of lytic *E*. *coli* BL21 (DE3) containing Mt-dUTPase; lane 4, effluents collected when loading protein samples onto His Trap chelating column; lanes 5–8, purified Mt-dUTPase, purified Mt-P79A-dUTPase, purified Mt-E126A-dUTPase, and purified human dUTPase. Western blot analysis using anti-His tag antibody is shown on the bottom. **(B)** Catalytic activity of Mt-dUTPase, Mt-P79A-dUTPase, Mt-E126A-dUTPase, and human dUTPase. The luminescence-based dUTPase activity assay was used. The time course experiments were repeated three times. **(C)** Schematic of principle for dUTPase assay.

Mt-dUTPase can hydrolyze dUTP to dUMP in *M. tuberculosis*. First, we purified Mt-dUTPase. The 10× His-tagged Mt-dUTPase was successfully expressed in *E. coli* BL21 (DE3) using a recombinant plasmid pET16b-Mt-dUTPase. The soluble protein Mt-dUTPase was then purified by a Ni^2+^ HisTrap chelating column. Purification samples were separated by SDS-PAGE following with Coomassie blue staining. A clear single band of 17.8 kDa was shown in the SDS-PAGE results, which was close to the predicted molecular weight of the target protein (Figure 1A). Western blot analysis using the anti-His tag antibody confirmed that it was definitely the Mt-dUTPase (Figure 1A).

When dUTPase hydrolyzes dUTP to dUMP, PPi is produced. As the byproduct of the catalytic reaction, PPi can trigger a luminescence reaction with the presence of the detection reagent containing AMP, luciferin, and luciferase (Figure 1C). As the output of PPi in the reaction increases, the luminescence signal will get stronger, which is positively correlated to the catalytic activity of dUTPase. Therefore, we quantified the catalytic activity of dUTPase indirectly by detecting the final luminescence of the reaction. Based on this, the catalytic activity of Mt-dUTPase at different times was detected. Figure 1B shows that the luminescence intensity (relative luminescence units, RLUs) of Mt-dUTPase assay gradually rose with the time increased (0–210 min), indicating a strong Mt-dUTPase activity.

### Inhibitory Effect of F0414 on *Mycobacterium tuberculosis* Deoxyuridine 5′-Triphosphate Nucleotidohydrolase

**FIGURE 2:**
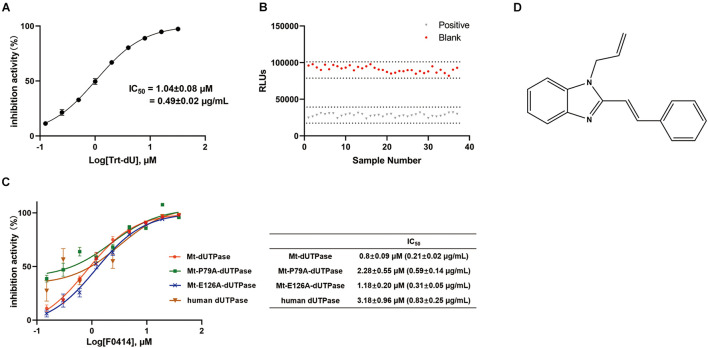
Evaluation and application of the screening assay. **(A)** Inhibition of Mt-dUTPase by the reference compound Trt-dU. Twofold dilutions (0.0625–16 μM) of Trt-dU were used. The activity of Mt-dUTPase treated by DMSO was regarded as 100%. **(B)** Z′ factor of the screening assay for Mt-dUTPase inhibitors. Mt-dUTPase was incubated with 20 μM reference compound Trt-dU or 2% DMSO for 1 h at room temperature. Then the luminescence intensity was detected and Z′ factor was calculated. **(C)** Dose-dependent inhibition of Mt-dUTPase, Mt-P79A-dUTPase, Mt-E126A-dUTPase, and human dUTPase by F0414. IC_50_ was plotted as the inhibition ratio of the luminescence intensity over the concentration of F0414 that fitted to a variable-slope dose-response equation. The experiments were repeated three times. **(D)** The structure of compound F0414.

To screen for inhibitors of Mt-dUTPase, we established the high-throughput screening model according to Mt-dUTPase *in vitro* activity assay. Trt-dU, a structure analog of dUTP, was used as a positive reference compound. The structure of Trt-dU is shown in Supplementary Figure 1. It exhibited a significantly inhibitory effect on Mt-dUTPase with an IC_50_ of 1.04 ± 0.08 μM (0.49 ± 0.02 μg/ml) (Figure 2A). The Z′ factor of the screening model was 0.90, suggesting an excellent repeatability and credibility of the assay (Figure 2B).

We screened 5,000 compounds in the initial primary screening to find the inhibitors of Mt-dUTPase. In total, 45 positive compounds showed more than 50% inhibition rate at a concentration of 10 μM. The positive rate for the initial primary screening was about 1%. Then, compounds that exhibited luminescence background inhibition or cytotoxicity were excluded. Finally, compound F0414 was selected to inhibit the Mt-dUTPase activity in a dose-dependent manner, with an IC_50_ of 0.80 ± 0.09 μM (0.21 ± 0.02 μg/ml) (Figure 2C). The structure of F0414 is shown in Figure 2D.

### Anti-tuberculosis Activity of F0414

For the essential role of Mt-dUTPase in *M. tuberculosis*, compounds that inhibit Mt-dUTPase activity might prevent the growth of *M. tuberculosis*. Therefore, the anti-TB activity of compound F0414 was determined by MABA method. The first-line anti-TB drugs rifampicin and isoniazid were used as the reference drugs. For *M. tuberculosis* standard strain H37Rv, the MIC of F0414 was 4 μg/ml. The MICs of F0414 for the clinical sensitive strain STB-FJ05349 and STB-FJ05060 were both 4 μg/ml. For the clinical multidrug-resistant strain MDR-FJ05120 and MDR-FJ05189, the MICs of F0414 were 8 and 2 μg/ml, respectively, which were significantly lower than that of rifampicin. For the clinical extensive drug-resistant strain XDR-FJ05195 and XDR-XZ06008, F0414 showed the inhibition with the MICs of 2 and 8 μg/ml, respectively, presenting better activities compared with rifampicin and isoniazid (Table 1). All the results indicated that compound F0414 possessed a potent anti-TB activity, with more effective inhibition on drug-resistant TB than rifampicin and isoniazid.

**TABLE 1:** Anti-TB activity of compound F0414.

3*Strain	**MIC (μg/ml)**		
	**F0414**	**Rifampicin**	**Isoniazid**
H37Rv	4	0.25	0.25
STB-FJ05349	4	0.5	0.5
STB-FJ05060	4	0.5	0.5
MDR-FJ05120	8	>256	4
MDR-FJ05189	2	>256	<0.5
XDR-FJ05195	2	4	>256
XDR-XZ06008	8	>256	>256

*H37Rv, *Mycobacterium tuberculosis* standard strain; STB, *Mycobacterium tuberculosis* sensitive strain; MDR, *Mycobacterium tuberculosis* multidrug-resistant strain; XDR, *Mycobacterium tuberculosis* extensive drug-resistant strain.*

### Molecular Docking Between F0414 and *Mycobacterium tuberculosis* Deoxyuridine 5′-Triphosphate Nucleotidohydrolase

**FIGURE 3:**
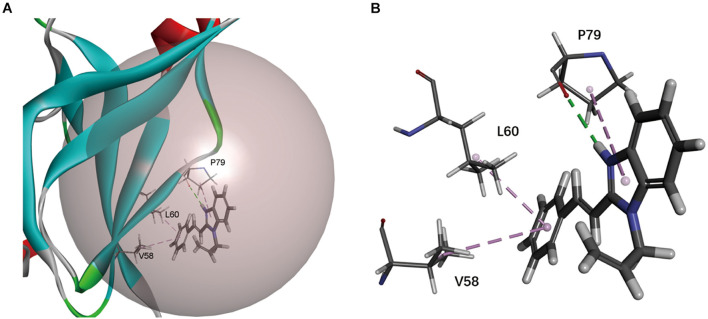
Molecular docking of Mt-dUTPase and F0414. **(A)** Overview of the active pocket of Mt-dUTPase bound to F0414. The active pocket is shown in gray ball. F0414 is represented by a stick model (light gray, hydrogen atoms; deep gray, carbon atoms; and blue, nitrogen atoms). **(B)** The detailed intermolecular bonds between Mt-dUTPase and F0414. The conventional hydrogen bond between the oxygen atom of P79 and hydrogen atom of F0414 is in green. The Pi-Alkyl bonds are shown in purple, which represented the interactions between V58 and the benzene ring of F0414, L60 and the benzene ring of F0414, P79, and the pyrrole ring of F0414.

To find the inhibitory mechanism of F0414 on Mt-dUTPase, we performed molecular docking between F0414 and Mt-dUTPase by Discovery Studio 2018R2 software. The optimal binding mode of F0414 and Mt-dUTPase with the highest score of −CDOCKER energy is shown in Figure 3A. F0414 occupied in the active center of Mt-dUTPase. The conventional hydrogen bond was formed between the oxygen atom of P79 and the hydrogen atom of F0414. The imidazole ring of F0414 can also interact with P79 by Pi-Alkyl bond, whereas two Pi-Alkyl bonds could be formed by the benzene ring of F0414 with L60 and V58 as well (Figure 3B). Since hydrogen bonds play a critical role in the binding of small molecules and proteins, we speculated that P79 was the key amino acid determining the interaction of F0414 and Mt-dUTPase.

### Inhibitory Effect of F0414 on *Mycobacterium tuberculosis* Deoxyuridine 5′-Triphosphate Nucleotidohydrolase Mutants

Based on the docking results, P79 played an essential role for the binding of F0414 and Mt-dUTPase. To analyze the inhibitory mode thoroughly, we mutated P79 to A79. Meanwhile, E126, which is located outside of the Mt-dUTPase active center, was mutated to A126 for the control. They were named as Mt-P79A-dUTPase and Mt-E126A-dUTPase, respectively. Both Mt-P79A-dUTPase and Mt-E126A-dUTPase were successfully purified and identified using the same methods mentioned previously (Figure 1A). SDS-PAGE and western blot using anti-His tag antibody showed the bands of approximately 17.8 and 17.7 kDa, respectively.

The *in vitro* catalytic activity of the mutant proteins was examined (Figure 1B). As expected, the catalytic activity of Mt-E126A-dUTPase remained almost the same as Mt-dUTPase, with only a little decrease. At the same time, the Mt-P79A-dUTPase mutant showed a diminution of about 40% in enzyme activity, which might be caused by the mutation of the key amino acid in the active center.

We next detected the inhibition effects of F0414 on the mutant proteins. F0414 showed the inhibition on Mt-P79A-dUTPase with an IC_50_ of 2.28 ± 0.55 μM, about three times higher than that of Mt-dUTPase (IC_50_ = 0.8 ± 0.09 μM). However, the IC_50_ of F0414 to Mt-E126A-dUTPase was 1.18 ± 0.20 μM, almost the same as it was for Mt-dUTPase (Figure 2C). Thus, the inhibitory effect of F0414 to Mt-dUTPase decreased obviously when the key amino acid P79 changed. We can infer that P79 of Mt-dUTPase played an essential role in the binding of F0414 to Mt-dUTPase.

### Inhibitory Effect of F0414 on Human Deoxyuridine 5′-Triphosphate Nucleotidohydrolase

Since Mt-dUTPase was used for drug screening, we speculated that compound F0414 might show weak inhibition for human dUTPase. Thus, we purified human dUTPase for the specificity examination of F0414. Human dUTPase was purified and identified as described previously, and a clear single band about 17.4 kDa was shown in the SDS-PAGE and western blot (Figure 1A). Then the activity of human dUTPase was determined, with an obvious increase compared with Mt-dUTPase (Figure 1B). The IC_50_ of F0414 to human dUTPase was 3.18 ± 0.96 μM, nearly fourfold higher than that of Mt-dUTPase (Figure 2C). This indicated that F0414 had a weaker inhibitory effect on human dUTPase, which also meant F0414 exhibited a selectivity to Mt-dUTPase.

### The Interactions Detected by Surface Plasmon Resonance Between F0414 and *Mycobacterium tuberculosis* Deoxyuridine 5′-Triphosphate Nucleotidohydrolase

**FIGURE 4:**
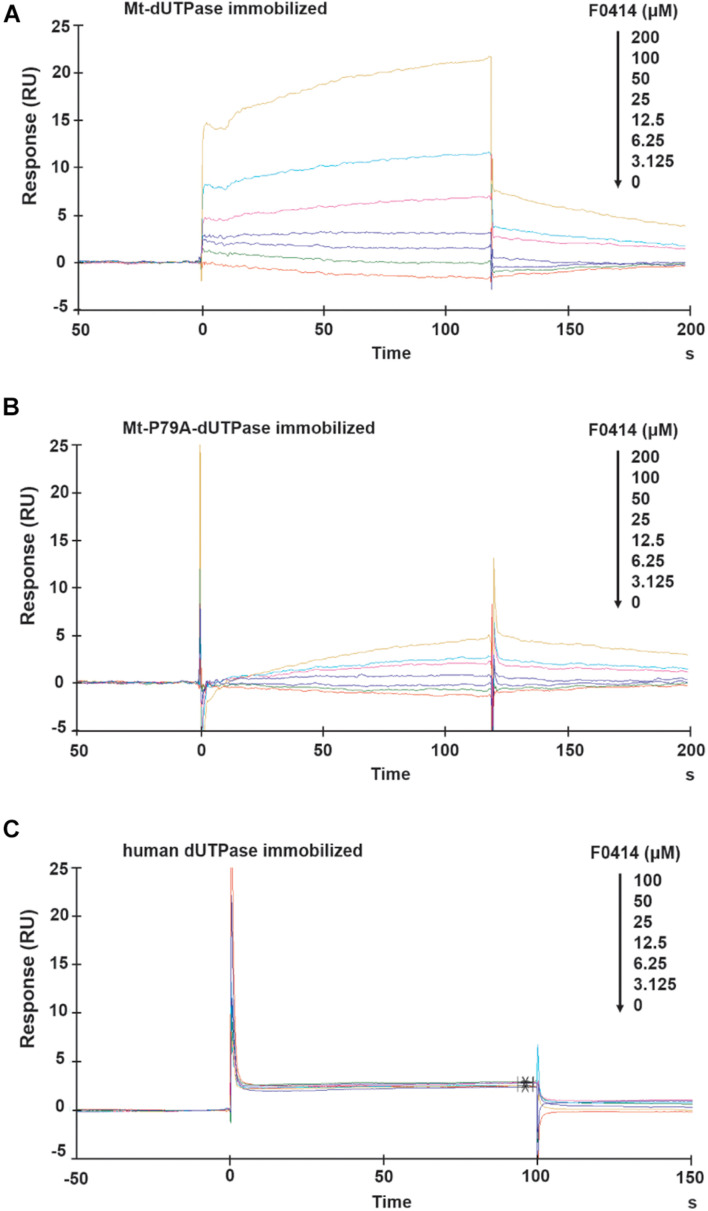
Surface plasmon resonance (SPR) analysis of the affinity between F0414 and dUTPase. **(A)** Binding of F0414 to Mt-dUTPase. Various concentrations (0–200 μM) of F0414 were injected into the chamber of a Mt-dUTPase coated CM5 sensor chip. The change of RU is shown. **(B)** Binding of F0414 to Mt-P79A-dUTPase. **(C)** Binding of F0414 to human dUTPase.

The SPR analysis was performed to further confirm the affinity between F0414 and Mt-dUTPase. When Mt-dUTPase was coated onto the sensor chip, F0414 could bind with it in a dose-dependent manner at concentrations of 0–200 μM (Figure 4A). The equilibrium dissociation constant (*K*_*D*_) was 2.877 × 10^–4^ M. Then, the sensor chip was immobilized with Mt-P79A-dUTPase. The binding result indicated that F0414 possessed a much weaker affinity with Mt-P79A-dUTPase (Figure 4B). Thus, we confirmed that P79 of Mt-dUTPase was the key amino acid for the interaction of F0414 with Mt-dUTPase.

The interaction between F0414 and human dUTPase was also analyzed by SPR experiment. As was shown in Figure 4C, when human dUTPase was coated on to the sensor chip, we observed almost no RU signals. This is in accordance with the preliminary conclusion that F0414 owned a considerable high selectivity and specificity to Mt-dUTPase.

### Cytotoxicity of F0414

**FIGURE 5:**
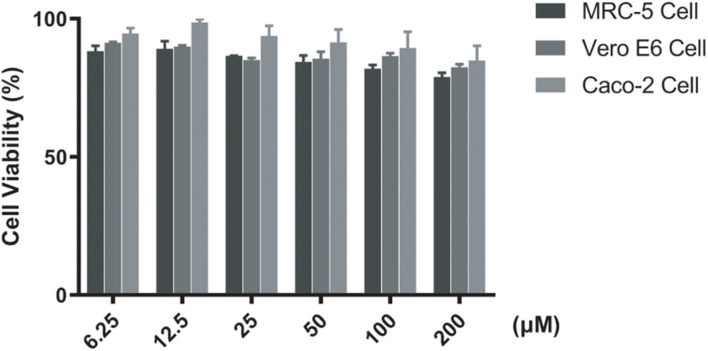
Cytotoxicity of F0414 measured by CCK-8 assay. MRC-5, Vero E6, and Caco2 cells were treated with F0414 from 6.25 to 200 μM.

The cytotoxicity of F0414 was performed on MRC-5, Vero E6, and Caco2 cells. All the CC_50_s (50% cytotoxic concentration) of F0414 to these cells were >200 μM (about 52 μg/ml), which were significantly higher than the MICs of its anti-TB activity (Figure 5). The results suggested a comparable selectivity of F0414 to *M. tuberculosis*.

## Discussion

Key enzymes in the biosynthesis of nucleotides are attractive drug targets for novel antibacterial agents (Rasouly and Nudler, 2018; Greene et al., 2020). For *M. tuberculosis*, several approved TB drugs are targeting the essential enzymes in DNA metabolism, including DNA replication and repair. For example, fluoroquinolones are used as second-line anti-TB drugs interfering with DNA topoisomerase and DNA gyrase (Pranger et al., 2019). Current researches placed emphasis on the key enzymes involved in the purines and pyrimidine metabolism in *M. tuberculosis*, including orotate phosphoribosyltransferase (OPRT), 5-phosphorybosyl-1-pyrophosphate synthase (PrsA), 5′-monophosphate dehydrogenase (GuaB2), thymidylate synthase ThyX, and ThyA (Donini et al., 2017a,b; Sahu et al., 2018; Sarkar et al., 2020). Compound VCC234718, DPU-2, and DPU-3 were identified as the anti-TB hits that targeted the aforementioned enzymes, with a remarkable selectivity toward human cell lines (Usha et al., 2011; Singh et al., 2017). Based on these, dUTPase, the essential enzyme for synthesis of dUTP, has been regarded as the potential drug target against *M. tuberculosis*.

As we know, dUTPase can be classified into three distinct families, which shows different characteristic forms as monomers, homodimers, and homotrimers (Persson et al., 2001). Most dUTPase, including that of prokaryote, eukaryote, and retroviruses, are all homotrimeric proteins, which are characterized by five conserved sequence motifs (Persson et al., 2001; Tarbouriech et al., 2005). The active sites lie at the interface of the adjacent units that are made up from the highly conserved motifs. Besides, the monomeric dUTPase is only found in Herpesviruses, such as Epstein–Barr virus (EBV) (Tarbouriech et al., 2005). It is encoded by a single polypeptide, which is longer than the sequence of the homotrimeric dUTPase and folds structurally emulating its active sites. It is reported that the EBV-encoded dUTPase could control the immune system by inducing the proinflammatory cytokines, playing a regulatory role (Ariza et al., 2013; Williams et al., 2019). In contrast, the dimeric dUTPase is identified in protozoa, like *Trypanosoma cruzi*, *Leishmania major*, and the bacterium *Campylobacter jejuni*, possessing the five characteristic motifs totally different from that presented in the trimer dUTPase (Harkiolaki et al., 2004; Moroz et al., 2004; Hemsworth et al., 2011). Due to the structural difference and relatively low sequence similarity of dimeric dUTPases with its human ortholog, dUTPases have been widely used for antiparasitic drug design (Nguyen et al., 2005).

Since both human and *M. tuberculosis* belong to the homotrimer dUTPase family, it might be an important concern in identifying Mt-dUTPase inhibitors without cross-reactivity with human dUTPase. Although the crystal structure of Mt-dUTPase is largely similar with human dUTPase, there are some specificities for Mt-dUTPase that is essential for its inhibitor design. It has been found that the C-terminal of Mt-dUTPase included a five-residue-loop region, which only existed in *M. tuberculosis*. The five-residue loop (E132-A136) changed the peptide chain folding that might be used as docking surface for inhibitors (Varga et al., 2008). This specific characteristic was of much significance, which might allow less reactivity with human dTUPase. Besides, there was another significant difference in the uracil binding active site of Mt-dUTPase that sets it apart from human dUTPase. In human dUTPase, the hydrogen bond donor for O4 of uracil was a backbone amide nitrogen atom of G76. However, in Mt-dUTPase, two amino acids S78 and P79 inserted and replaced the nitrogen atom of N77 (the same function with G76 of human dUTPase) sidechain in the position of hydrogen bond donor for O4 (Chan et al., 2004). The insertion of two amino acids significantly changed the mode of nucleotide binding pocket. It might be effective to exploit the selective drugs based on differences between binding pockets of human dUTPase and Mt-dUTPase. In this research, we found that P79 was the key amino acid for the binding of compound F0414 and Mt-dUTPase, which was absent in human dUTPase. It might be the reason that F0414, with potent anti-TB activity, was low toxic to human cell lines. F0414 has been the first anti-TB compound targeting Mt-dUTPase, which also has a selectivity for human dUTPase.

In this study, two approaches have been used to evaluate the binding of F0414 and dUTPase (including Mt-dUTPase, Mt-P79A-dUTPase, and human dUTPase). In the luminescence-based dUTPase activity assay, the IC_50_s for F0414 to inhibit Mt-dUTPase, Mt-P79A-dUTPase, and human dUTPase were 0.80 ± 0.09, 2.28 ± 0.55, and 3.18 ± 0.96 μM, respectively. When SPR method was applied, the binding strength of F0414 with Mt-dUTPase, Mt-P79A-dUTPase, and human dUTPase gradually decreased as was shown by RU values. The two approaches gave the same conclusion that P79 was the key amino acid for the interaction of F0414 to Mt-dUTPase. Moreover, compound F0414 had weak interaction with human dUTPase, which might be the reason for its low cytotoxicity. As we know, IC_50_ and *K*_*D*_ are inhibitory and binding parameters to evaluate the inhibitory potencies and/or binding strengths for interactions of compounds and proteins (Bigott-Hennkens et al., 2008). However, we only reported the *K*_*D*_ for F0414 on Mt-dUTPase in this research. The *K*_*D*_s for F0414 on Mt-P79A-dUTPase and human dUTPase could not be correctly calculated for both the association and dissociation phases did not follow the typical SPR profiles.

F0414 is a benzimidazole compound with no biological activities reported. There has been a lot of benzimidazole compounds used in clinic, which exhibited various properties, such as anti-tumor, anti-viral, anti-bacterial, anti-fungal, and anti-metabolic syndrome (He et al., 2019; Shi et al., 2019; Singh et al., 2019; Tharmalingam et al., 2019). For example, pracinostat, a novel benzimidazole-based histone deacetylase inhibitor, has been identified as a potential agent for treating breast cancer (Chen et al., 2020). Samatasvir, a selective inhibitor of HCV replication *in vitro* with picomolar activity, was also one of the benzimidazole derivatives that combine with other antiviral drugs in the clinical therapy of HCV-infected patients (Bilello et al., 2014). Ridinazole, another benzimidazole compound, was used as a novel antibacterial agent against *Clostridium difficile* (Cho et al., 2019). Besides, the benzimidazole-based scaffold has been shown to have anti-mycobacterial activity, making it a promising structure for the discovery of new anti-TB agents (Keri et al., 2016; Sirim et al., 2020; Parwani et al., 2021). However, there were no biological activities reported on F0414 in the previous studies. Based on our research, F0414 has the potential to be an anti-TB hit compound.

## Conclusion

In summary, we found a novel anti-TB agent F0414 that targeted Mt-dUTPase. F0414 is the first compound reported to inhibit Mt-dUTPase by binding with P79 that is absent in human dUTPase. Combined with the significant anti-TB activity weak effect on mammalian cells, we believe that compound F0414 might have the potential for anti-TB drug discovery. In the future, we hope to find more potent Mt-dUTPase inhibitors through chemical modification of F0414 with stronger anti-TB activity and less toxicity.

## Data Availability Statement

The original contributions presented in the study are included in the article/Supplementary Material, further inquiries can be directed to the corresponding author/s.

## Author Contributions

J-DJ, SS, and YL planned and designed the study. YZ, HZ, YC, and LQ carried out the experiment and collected the data. YZ, HZ, and YH analyzed the data. YL, YZ, and J-DJ wrote and revised the manuscript. All authors approved the final version of the article.

## Conflict of Interest

The authors declare that the research was conducted in the absence of any commercial or financial relationships that could be construed as a potential conflict of interest.

## Publisher’s Note

All claims expressed in this article are solely those of the authors and do not necessarily represent those of their affiliated organizations, or those of the publisher, the editors and the reviewers. Any product that may be evaluated in this article, or claim that may be made by its manufacturer, is not guaranteed or endorsed by the publisher.
