# Supplementary figures and images for: Screening and Identification of a Novel Anti-tuberculosis Compound That Targets Deoxyuridine 5′-Triphosphate Nucleotidohydrolase
Source: Front Microbiol. 2021 Oct 11;12:757914. doi: 10.3389/fmicb.2021.757914 (PMC8544286; doi:10.3389/fmicb.2021.757914)

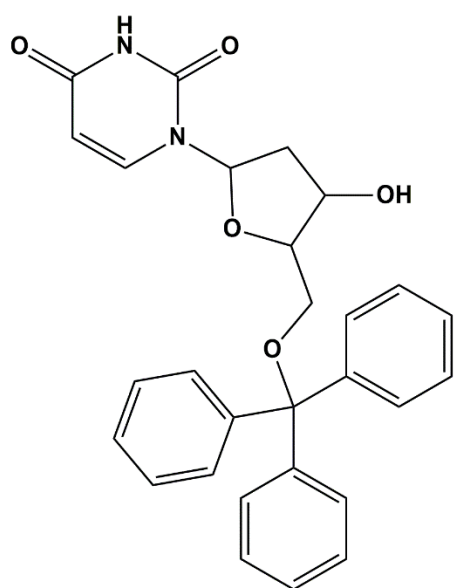

**Supplementary Figure 1.** The structure of Trt-dU.

Supplement: Supplementary file 1 [file Data_Sheet_1.PDF]
